# Uncovering the Role of Mindfulness in Autonomous Motivation across Physical Education and Leisure Time: Extending the Trans-Contextual Model

**DOI:** 10.3390/ijerph192012999

**Published:** 2022-10-11

**Authors:** Djenna Hutmacher, Melanie Eckelt, Andreas Bund, André Melzer, Georges Steffgen

**Affiliations:** 1 Department of Behavioural and Cognitive Sciences, University of Luxembourg, Campus Belval, 11, Porte des Sciences, L-4366 Esch-sur-Alzette, Luxembourg; 2 Department of Education and Social Work, University of Luxembourg, Campus Belval, 11, Porte des Sciences, L-4366 Esch-sur-Alzette, Luxembourg

**Keywords:** mindfulness, motivation, physical education, physical activity, leisure time

## Abstract

Mindfulness is assumed to foster the ability to consistently act in line with one’s authentic self; a skill which has been found to enhance students’ autonomous motivated behavior in the educational context. However, evidence regarding how mindfulness can be integrated into existing conceptual frameworks such as the trans-contextual model is scarce. Therefore, the present study aimed to evaluate the role of mindfulness in students’ autonomous motivation in the school and leisure time contexts. Overall, *N* = 1877 students (*M* = 14.74 years, *SD* = 2.63) indicated their self-reported mindfulness, their perceived need for support in physical education, their autonomous motivation during physical education and leisure time, as well as their perceived behavioral control, attitude, subjective norm, and intention toward physical activity. Physical activity was additionally measured physiologically for *n* = 240 students using accelerometers. Path model analyses revealed that the inclusion of mindfulness substantially improved the trans-contextual model fit. Perceived autonomy support positively predicted mindfulness, which, in turn, predicted autonomous motivation in physical education and leisure time, attitude, subjective norm, and perceived behavioral control. Furthermore, mediation analyses revealed the significant indirect effects of mindfulness on physiological and self-reported physical activity. Based on these results, mindfulness can be considered a key factor in fostering students’ motivation to become physically active.

## 1. Introduction

As education in the twenty-first century comes along with new challenges, the question of how skills and knowledge taught in schools can be integrated into the students’ everyday lives arises. The trans-contextual model [1] addresses this transfer by characterizing the processes of autonomous motivation in school as an important factor of behavioral engagement over a longer period, which, in turn, predicts autonomous motivation beyond the school context. The introduced model entails a multi-theoretical approach, combining self-determination theory (SDT) [2], Vallerand’s [3] hierarchical model of intrinsic and extrinsic motivation, and the theory of planned behavior (TPB) [4]. In accordance with SDT, the first key assumption of the trans-contextual model is that an autonomy supportive climate in class is associated with a greater perception of autonomous (self-determined) motivation in class [5]. Second, the authors propose that autonomous motivation toward leisure-time-based physical activities is predicted by autonomous motivation in physical education. In addition, autonomous motivation in an out-of-school context predicts the attitude, self-perceived behavioral control, and subjective norms toward physical activity, which, in turn, predict intentions toward, and actual engagement in, out-of-school physical activity. Overall, the presented model displays an important framework for fostering an understanding of how students may transfer their autonomous motivation from the school context toward their everyday lives [5,6].

### 1.1. Self-Determination Theory (SDT)

As a key aspect of the trans-contextual model, SDT is an empirically driven theory of human motivation and development, treating motivation on a continuum of behavioral regulation [7]. Differentiating between controlled (more externally regulated forms of motivation) and autonomous motivation (more self-determined regulations of motivation), the transition from a more controlled toward a self-reflective behavior integrated into one’s self is called “internalization” [8]. Regarding autonomous motivation, which reflects engaging in a behavior for reasons of volition to obtain self-referenced outcomes, three different regulation types are presented in SDT [7]. Firstly, *identified regulation* reflects the engagement in a behavior, which serves as a purpose for a self-endorsed outcome, which is personally important. Secondly, *integrated regulation*, a more self-regulated form along the continuum of SDT, occurs when identified regulations are entirely assimilated to the self (internalized), meaning that the personally important values of the behavioral outcomes are fully integrated into the sense of self. Finally, *intrinsic motivation*, the most self-determined (autonomous) behavioral regulation form, represents the engagement in an activity for the intrinsic (inherent) satisfaction and enjoyment of the activity itself. In contrast, two controlled types of motivation are described in SDT. As such, *external regulation* reflects the most controlled form of behaviors, relying entirely on external drivers, such as the receipt of a reward or the avoidance of punishment. Finally, *introjected regulation* describes a more internalized but still externally pressured behavioral regulation, undertaken in order to avoid possible feelings of shame or guilt when not executing the behavior.

Internalized (i.e., autonomous) regulated forms of motivation have been found to be associated with the continuance of self-determined learning and activities in school, which lead to various positive outcomes, such as academic attainment, performance, knowledge, psychological well-being, and happiness [9,10,11]. In order to facilitate the “internalization” of autonomous motivation, SDT proposes the three innate psychological needs for *autonomy* (the sense of being the causal agent of one’s own behavior), *competence* (the sense of being capable to master a desired behavior), and *relatedness* (the sense of being connected and attached to significant others) [8]. According to existing research in the educational context, the support of these needs by the physical education teacher ameliorated their students’ satisfaction of their needs, which was found to be directly related to a more internally motivated regulation [12,13,14]. Thus, in extension to the first assumption of the trans-contextual model, we assume that it is not autonomy support alone, which plays a role in predicting autonomous motivation in physical education. Instead, we expect competence and relatedness support to be further significant predictors of autonomous motivation, as proposed by SDT.

With regard to Vallerand’s hierarchical model of intrinsic and extrinsic motivation [3], it is claimed that similar motivational types are interrelated between different contexts, and on the hierarchical level, towards similar global, as well as situational, motivation types. Hagger and colleagues [1] therefore assumed, and confirmed that, in their trans-contextual model, students’ autonomous motivation in the physical education context was related to their autonomous motivation level for leisure-time-based physical activity.

### 1.2. Theory of Planned Behavior

As a further assumption of the trans-contextual model, self-regulated behavior (i.e., autonomous motivation) influences the intention to become physically active, and is mediated by the proposed constructs of the theory of planned behavior toward a specific behavior [4]. The theory of planned behavior posits that human behavior is guided by three separable components reflecting beliefs about self-efficacy. Firstly, beliefs of the consequences or attributes of the behavior refer to a favorable or unfavorable *attitude* toward the behavior. Secondly, beliefs about factors that may promote or hinder performing the behavior are called *perceived behavioral control*, which could be used as a measure for the actual control of the behavior in question (i.e., if people are realistic in their judgments of behavior difficulty) [15]. Thirdly, beliefs about the normative expectations of others may result in social pressure or *subjective norms*, which further influence the intention to carry out the behavior. *Intention*, that is, the willingness to engage in a behavior, has been found to be the immediate antecedent of behavior, formed by the three components of attitude, perceived behavioral control, and subjective norms [16].

The theory of planned behavior has been supported in several recent empirical endeavors from a variety of scientific domains, including health behavior [17] and teaching in education [18], or, more specifically, physical activity [19]. However, meta-analyses revealed that, on average, intentions explain no more than 30% of subsequent behaviors [20,21]. This so-called intention–behavior gap in predicting behavior [22], could be extenuated by additionally improving memory, and by creating habits and automatic processes to initiate behavior [23,24,25,26].

### 1.3. Conceptual Integration of Mindfulness into the Trans-Contextual Model

Although the trans-contextual model demonstrates robust theoretical and scientific evidence, it is restricted to the implication of a need-supportive climate in class to promote autonomous motivation in the long term [1,5]. Therefore, in the present study, another important mental state, namely mindfulness, is evaluated, as it may influence said motivational promotion, alongside the satisfaction of the basic psychological needs in class. As described in the work of Kabat-Zinn [27,28], mindfulness can be defined as a non-judgmental awareness of the present moment with all senses. It is specified as the practice of consciously bringing back one’s attention to the “here and now”. Mindfulness has been proven to provide a climate of reduced stress [29], and improves well-being [30] and engagement in class [31], thus representing a promising concept to be considered in the educational context. As such, the advantages of implementing mindfulness in education have been demonstrated in numerous studies on evidence-based programs aimed at boosting social competent behaviors, more positive self-concept, optimism [32], improvements in working memory, attention, academic skills, emotional regulation [33], moral reasoning, and ethical decision making [34], in both elementary and high school [35]. In the context of SDT, awareness is furthermore viewed as a significant component of self-regulated behavior and well-being [36]. Overall, the question of whether higher self-reported mindfulness and behavioral self-regulation in school have an impact on related out-of-school physical activity is of considerable importance. This is especially relevant as only around 30% of adolescents across 146 countries reach the recommended World Health Organization (WHO) guidelines of being physically active for at least 60 min a day [37].

Although mindfulness programs and practices in education have provided promising results, there appears to be considerable heterogeneity with regard to their significance, acceptance, and theoretical background in existing research [38]. Additionally, the authors state that such programs are often based on exploratory non-consensus frameworks with few replication studies. Thus, more research is needed in order to establish a profound school-based framework for interventions in educational contexts incorporating mindfulness. In this study, we wish to address this issue by investigating the relationship between mindfulness and mechanisms whose educational relevance has already been established; more specifically, to the different constructs of the trans-contextual model [1].

When considering the significant role of mindfulness within the school context, we expect autonomy support of the physical education teacher to play a key role in predicting mindfulness. Furthermore, we assume autonomy support to be related to autonomous motivation and mindfulness. For instance, if the physical education teacher encourages students to take control of their own actions (e.g., choosing the adequate exercise matching one’s performance ability), this fulfillment of their need for autonomy may not only lead to a higher perception of agency [39], but also to increased self-awareness [40]. Consequently, we suspect that students’ increased self-awareness and perceived autonomy may inherently increase their perceived overall mindfulness, leading to behaviors that are congruent with their own experienced self.

Furthermore, as self-regulation requires explicit awareness [41], and as SDT treats awareness as an essential aspect for the internalization of a self-determined regulation, we additionally assume that mindfulness is directly related to autonomous motivation. For example, while integrated regulation represents a form of highly autonomous motivation, it requires self-reflection and the awareness to recognize if one’s own values and needs reflect one’s actions [8]. Thus, mindfulness functions as a prerequisite to the awareness of whether one is consistently acting with one’s authentic self [42] by being aware of external circumstances, which may affect personal regulatory processes [43]. In their meta-analysis, Donald and colleagues [44] obtained scientific evidence for this relationship, in the sense that mindfulness was directly associated to intrinsic motivation and identified regulation.

Even if the conceptual alliance between SDT and mindfulness is substantial, available scientific evidence with regard to how mindfulness may be integrated into the mechanisms of SDT is scarce and generally lacking in the trans-contextual model. So far, existing research has been limited to findings showing that awareness is related to self-regulation and mindfulness, and, in turn, that self-reported mindfulness is associated with autonomous, self-regulated behavior [44,45,46]. Given the promising nature of mindfulness in extending SDT, the present study investigates how mindfulness complements the mechanisms of the trans-contextual model. We therefore assume that the support of autonomy by the physical education teacher in class will positively affect students’ self-reported mindfulness, which, in turn, will positively affect their autonomous motivation (i.e., intrinsic motivation and identified regulation) in both physical education and leisure-time-based physical activity.

As a particularity of the trans-contextual model, it has furthermore been found that an increased autonomous motivation toward physical activity in leisure time is directly related to subjective norms, perceived behavioral control, and attitude [1]. In accordance with these findings, awareness, attention, and autonomous motivation are considered crucial for an individual to monitor inner experiences and environmental factors in order to evaluate the current situation as it is experienced [42]. In this sense, it is expected that mindfulness might be directly related to the three antecedents of the intention to become physically active, as proposed by the theory of planned behavior.

Importantly, the underlying concept of mindfulness considers awareness only independently of the contents of the mind [47]. In contrast, however, the TPB suggests that *attitudes* are crucial for the formation of a behavioral intention, which denotes a mental state comprising moods, associations, expectations, and intentions. In contrast to Western academic psychological definitions of mindfulness, including attitude [48], we agree with Mikulas [47] that this addition could lead to confusion in the understanding and application of mindfulness, as the concept of mindfulness primarily considers a non-judgmental awareness in a given situation. In this sense, we believe that the concepts of attitudes and mindfulness should be treated as distinct entities. Therefore, we assume that mindfulness has an impact on attitudes with regard to social behavior. For example, Langer and colleagues [49] found that sixth graders without disabilities who participated in mindfulness training showed higher attendance rates and increased teammate choices of children with disabilities. Objective self-awareness (e.g., self-awareness promoted through manipulations such as mirrors or TV cameras), has additionally been found to be relevant for attitude–behavior consistency [50].

Similarly, according to the concept of *perceived behavioral control*, Pagnini and colleagues [51] propose a framework to include mindfulness, as both concepts have similar positive outcomes on physical health and psychological well-being. When individuals are aware of their current situation, they can adapt to changes more quickly, resulting in experiencing control. In their experimentally created mindfulness-inducing Rejection Behavioral Monitoring Technique, Perlmuter and Langer [52] furthermore found that control could be effectively enhanced through focusing on rejected alternatives related to a specific activity. Accordingly, mindfulness has been found to positively affect perceived behavioral control [41,53].

In contrast to perceived behavioral control and attitudes, research on the conceptual integration of mindfulness to subjective norms is scarce. However, it has been found that mindfulness is positively associated with expressing oneself in social situations through engaging in empathic behavior, while at the same time leading to a decrease in social anxiety [54]. Furthermore, mindfulness-based interventions improve prosocial responding in both adults [55] and adolescents [56]. Thus, it is expected that mindfulness will be related to the students’ perception of subjective norms due to an increased awareness.

Moreover, mindfulness has been found to moderate the relationship between intention and physical activity in leisure time [57]. For instance, more mindful individuals were better able to control the influences of counter-intentional habits and thoughts, perhaps due to higher attention and awareness of their inner experiences and routines [57]. Therefore, an indirect effect of mindfulness on intention and behavior is expected in the presented study.

As a particularity of the present study, it needs to be stressed that it is unique in conceptually integrating mindfulness into the trans-contextual model. At the same time, this study also represents an attempt to test the trans-contextual model on both self-reported physical activity behavior and physiologically measured physical activity via accelerometers. As self-report and physiological measures on physical activity show low to moderate correlation values, it is of particular interest to include and compare the results from both of these instruments of measurement [58].

### 1.4. The Present Study

Taken together, the present study aims at integrating the concept of mindfulness into the trans-contextual model. As mindfulness-based intervention programs have proven beneficial for students in class [35], and as the trans-contextual model provides evidence for a better understanding of the contextual transfer of self-regulated behavior [5], we assume that integrating mindfulness will provide an important conceptual benefit for the trans-contextual model. Specifically, we expect that the support of autonomy by the physical education teacher predicts mindfulness, while, at the same time, mindfulness should positively predict autonomous motivation in physical education and leisure time. Additionally, we hypothesize that mindfulness is positively related to attitude, subjective norm, and perceived behavioral control. Finally, mindfulness is suspected to be indirectly related to intention and both to self-reported and physiologically measured physical activity behavior, mediated by attitude, subjective norm, and perceived behavioral control.

## 2. Methods

### 2.1. Participants

A total of 1877 students (*M* = 14.74 years old, *SD* = 2.63; 955 males (50.9%)) aged between 10 and 23 years from nine primary and five secondary schools in Luxembourg participated in this study (Please note that different parts of the dataset used in the present study have also been investigated in other publications [6,59]. However, the dataset was incorporated in the present study in order to address unique and different research questions, analyses, and findings). The 14 schools (134 classes) were selected randomly via stratified sampling (i.e., controlling for the geographical regions; North, South, Center, East, representing most schools in Luxembourg) by the SCRIPT (Service de Coordination de la Recherche et de l’Innovation Pédagogiques et Technologiques), an entity of the Ministry of National Education, Childhood and Youth in Luxembourg. Overall, 382 students (20.4%) attended elementary school (grades 5 and 6). Of the remaining students attending secondary school, 436 (23.2%) attended grade 7, while 522 (27.8%) attended grade 9, and 537 (28.6%) grade 11. In addition to self-report measurements and for analyzing physical activity physiologically via accelerometers, 312 students of the total sample volunteered to also wear an accelerometer over seven consecutive days. Data from 240 students (*M* = 13.50 years old, *SD* = 2.61; 104 males (43.3%)) ranging from 10 to 20 years from four primary and five secondary schools were included in the additional analyses of the accelerometer measurements. The remaining students had to be excluded from the physiologically measured physical activity data, as they did not provide valid accelerometer measures from at least eight hours over four days including one weekend day [60].

### 2.2. Measures (Please Note That Several Items Were Used for the Study as Presented in Hutmacher and Colleagues [60], but Are Listed Here in Detail for a thorough Understanding of the Study Design)

The scales used in the present study (except for the mindfulness scale) have been translated from English into French and German by two psychologists (using the back-translation technique) [61].

### 2.2.1. Constructs of the Self-Determination Theory (SDT)

In order to assess the participants’ perceived *need support* for autonomy, competence, and relatedness of the physical education teacher, overall, 16 items were used, based on the study items of Standage and colleagues [62]. Responses to all items were preceded by the stem “in this physical education class…” and rated on a seven-point scale (1 = not agree at all, 7 = totally agree). Seven out of sixteen items were used for autonomy support (α = .82; e.g., “the physical education teacher encourages me to ask questions”). Four items were used for competence support (α = .83; e.g., “the physical education teacher helps me to improve”), and five items for relatedness support (α = .81; e.g., “the physical education teacher has respect for me”). Confirmatory factor analyses revealed a good model fit for the three factors, χ^2^ = 1226.283; *df* = 101; *p* < .001; RMSEA = .077; 90% CI = [.073; .081]; SRMR = .04; CFI = .93; TLI = .91, with factor loadings ranging between .46 and .77. The revised Perceived Locus of Causality Scale (PLOC-R) [63] was used to measure the *autonomous motivation in physical education*. For the present study, students answered two scales following the stem “I participate in physical education …” using a seven-point scale (1 = not agree at all, 7 = totally agree). Each subscale consists of four items for identified regulation (α = .85; e.g., “Because it is important to me to do well in physical education”) and intrinsic motivation (α = .82; e.g., “Because physical education is exciting”). Good psychometric properties have been found for this instrument, as reported in Hutmacher and colleagues [64]. The Behavioral Regulation in Exercise Questionnaire (BREQ-II) [65] was used to assess the *autonomous motivation in leisure time*. Students provided their answers on a seven-point scale (1 = not agree at all, 7 = totally agree). Only the subscales of identified regulation (α = .76; e.g., “It’s important to me to exercise regularly”) and intrinsic motivation (α = .87; e.g., “I enjoy my exercise sessions”), each comprising four items, were used in the present study. Good psychometric properties have been obtained for this questionnaire (please see Hutmacher and colleagues [64]).

### 2.2.2. Constructs of the Theory of Planned Behavior (TPB)

The present study used 16 items from Hagger and colleagues [1], which were created according to the procedure proposed by Ajzen and Madden [66]. The *attitude* to physical activity (α = .90) was measured with seven bipolar adjectives (e.g., harmful–beneficial) that were introduced by “I find being physically active in my free time for at least 60 min a day...”. All adjectives were rated on a seven-point semantic differential scale, with higher values representing the positive adjective. The *perceived behavioral control* (self-assessed ability) of students to regularly be physically active in their free time was recorded using three items (α = .77). Two items (e.g., “I have full control over whether I am active in my free time for at least 60 min a day”) were rated on a seven-point scale (1 = not agree at all, 7 = totally agree). The item “How much control do you have about being physically active in your free time for at least 60 min a day” was rated on a seven-point scale from one (“no control at all”) to seven (“complete control”). To assess the *subjective norm*, three items based on the injunctive norm (e.g., “People who are important to me encourage me to be physically active in my free time”) were used. These items showed an internal consistency of α = .72 and were rated on a seven-point scale (1 = not agree at all, 7 = totally agree). Two items were used to measure the *intention* to become physically active (α = .79). The first item, “I intend to be physically active for at least 60 min a day for the next 5 weeks”, was rated on a seven-point Likert scale (1 = not agree at all, 7 = totally agree), whereas students answered “I intend to be physically active for at least 60 min a day with the following regularity” on an eight-point Likert scale (0 = never, 7 = daily).

### 2.2.3. Mindfulness

The fourteen-item short form of the Freiburg Mindfulness Inventory (FMI) [67], as well as the French translated version [68] were used (answers were indicated on a seven-point scale with 1 = not agree at all and 7 = totally agree). The mindfulness scale (e.g., “I see my mistakes and difficulties without judging them”) showed an internal consistency of α = .82.

### 2.2.4. Self-Report and Physiological Measurement of Physical Activity

The self-reported physical activity behavior during leisure time was assessed via a single item, “On how many days of a regular week are you physically active for at least 60 min?” on an eight-point scale (from 0 = never, to 7 = on each day). In order to assess the physiological physical activity, the accelerometer ActiGraph wGT3X-BT was used to measure the acceleration of the body in different spatial dimensions. Research has shown the good reliability and validity of this apparatus [69]. Please note that only the physical activity measured in the out-of-school context was used for the construct of physiological physical activity in subsequent analyses. In order to analyze the physiological physical activity (please refer to Section 2.4), the height of the students was taken via a measuring tape and their weight via a pair of scales.

### 2.3. Procedure

Data were collected over the course of about two months and digitally entered via self-reported questionnaires using the secured platform OASYS [70] from the University of Luxembourg during the first trimester of the participants’ school year in autumn. The self-reported scales were filled out in class on a computer or tablet under the continuous supervision of trained research assistants. Due to this digital testing approach, no missing data need to be reported, because the students were unable to complete the questionnaire if they had missed an item. The duration of questionnaire completion varied between 30 and 50 min, depending on the age of the students. All students whose physical activity was additionally measured physiologically, individually received detailed information about the accelerometer by trained personnel at school and during a school lesson. Each accelerometer was previously initialized at a 30 HZ frequency. Students were instructed to wear the accelerometer on the right hip for seven consecutive days. They were told to wear the accelerometer anytime while being awake, except for water-based activities. Additionally, participants’ height and weight were measured to further analyze the physical activity rates. After one week, the accelerometers were returned in class and potential ambiguities were clarified. All participants signed informed consent forms. Additional written permission was required from the legal representative of the participants younger than 16 years. Ethical approval was provided by the Ethics Review Panel of the University of Luxembourg.

### 2.4. Data Processing and Analysis

The ‘Statistical Package for the Social Sciences’ (SPSS; 25th version) software [71] was used for data transformation and descriptive analyses. IBM SPSS Amos 26 was used to perform the confirmatory factor analyses and path models. The univariate and multivariate distributions of the items and the scales were verified. The internal reliability and factorial structure revealed good psychometric properties. A minimum value of .40 was accepted for factor loadings [72]. Items belonging to a joint subscale were averaged for the use in correlation and path analyses. Inter-correlations (Pearson’s *r* and point-biserial correlations as effect sizes) were analyzed between the different scales. Model fit was evaluated, and a reasonable fit was accepted for the root mean squared error of approximation (RMSEA ≤ .08), its 90% confidence interval (CI), the standardized root mean square residual (SRMR ≤ .12), comparative fit index (CFI ≥ .90), and Tucker–Lewis index (TLI ≥ .90), as described by Hu and Bentler [73]. Good model fit cutoffs were considered as RMSEA ≤ .06, SRMS ≤ .08, CFI ≥ .95, and TLI ≥ .95 [74]. Given the sensitivity of the χ^2^–statistics to sample size, model fit assessment was primarily based on the remaining fit indices. According to the cut-off value criterion of Cheung and Rensvold [75], model comparisons were based on a ΔCFI (change in the Comparative Fit Index), representing a statistically significant model fit difference when ΔCFI > .01.

Accelerometer data were processed and analyzed using the software ActiLife v6.13.4 (Actigraph Inc., Pensacola, FL, USA). In order to generate the students’ average time spent in moderate to vigorous physical activity (MVPA) per day, the threshold counts from the calibration study by Evenson and colleagues [76] were used for the students from 10 to 18 years, and the threshold counts by Troiano and colleagues [77] for the students older than 18 years. The algorithm by Choi and colleagues [78] was used to identify the time spent not wearing the accelerometer.

## 3. Results

Referring to Table 1, the different correlation coefficients of the measured scales were found to load in the expected way, according to the theory of the trans-contextual model [5]. Similar scales correlated higher, as, for example, the three scales of the students’ perceived psychological need support of the physical education teacher (each *r* = .82). Furthermore, autonomous motivation in the context of physical education significantly and positively correlated with autonomous motivation in the leisure time context (*r* = .59). According to the theory of planned behavior [4], and as expected, attitude, subjective norm, and perceived behavioral control were positively related to the intention to be physically active, which, in turn, was positively related to both self-reported and physiologically measured physical activity. Mindfulness was positively and significantly related to all measured variables of the trans-contextual model. Participants reported an average of *M* = 3.20 (*SD* = 1.97) days of at least 60 min of MVPA per week. Overall, 8.3% of the adolescents reported being physically active for at least 60 min every day. The 240 students whose physical activity rates were also physiologically measured via accelerometers showed an average of *M* = 47.47 (*SD* = 19.60) minutes per day in moderate to vigorous physical activity (MVPA).

In order to test the different assumptions, four different path models were conducted. First, the basic model akin to the trans-contextual model was tested as Model 1a and revealed an acceptable model fit (please refer to Table 2 for all model fit indices of the four different path models.

**Figure 1:**
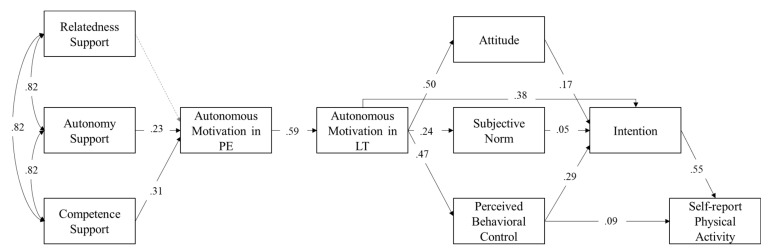
Path model (Model 1a) of the trans-contextual model. Model comprised a sample size of *N* = 1877 students. All calculated and standardized estimation paths are presented. Significant paths are depicted by solid arrows (*p* < .05), while non-significant paths are represented by dashed arrows (*p* > .05). PE = Physical education context; LT = Leisure time context.

The assumptions of the trans-contextual model were found as presented in Figure 1. However, beyond the assumption of the trans-contextual model, and in accordance with the SDT, competence support in physical education was, next to autonomy support, also significantly positively related to autonomous motivation in physical education. Nevertheless, for relatedness support, the expected positive relation to autonomous motivation in physical education turned out to be statistically insignificant (*p* > .05). Furthermore, autonomous motivation in physical education was positively related to autonomous motivation in leisure time, which, in turn, predicted attitude, subjective norm, and perceived behavioral control. Furthermore, perceived behavioral control was directly associated with self-report physical activity. However, this relationship was partially mediated by intention. Overall, Model 1a explained a considerable proportion of variance in all seven outcomes: autonomous motivation in physical education (*R*^2^ = .32), autonomous motivation in leisure time (*R*^2^ = .35), attitude (*R*^2^ = .25), subjective norm (*R*^2^ = .06), perceived behavioral control (*R*^2^ = .23), intention (*R*^2^ = .47), and self-report physical activity (*R*^2^ = .36).

**Figure 2:**
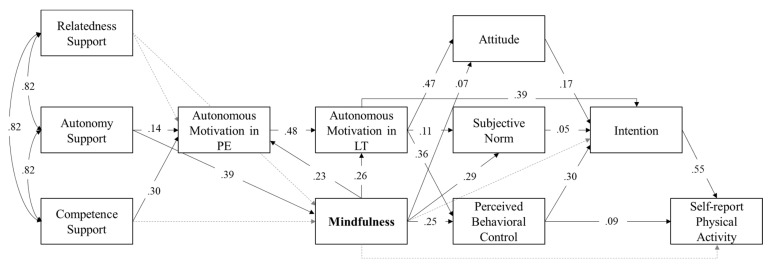
Path model (Model 1b) including mindfulness. Model comprised a sample size of *N* = 1877 students. All calculated and standardized estimation paths are presented. Significant paths are depicted by solid arrows (*p* < .05), while non-significant paths are represented by dashed arrows (*p* > .05). PE = Physical education context; LT = Leisure time context.

Secondly, as a further purpose of the present study, mindfulness was integrated into the trans-contextual model (Model 1b). Table 2 illustrates that additionally including mindfulness improved the fit of Model 1b, compared to Model 1a (*p* < .05; ΔCFI > .01). However, only the basic need for autonomy support was a significant and positive predictor of mindfulness, while both autonomy support and competence support were positive predictors for autonomous motivation in physical education (*p* < .05; please refer to Figure 2). Notably, relatedness support failed to predict both autonomous motivation in physical education and mindfulness. Autonomous motivation in physical education was, as expected, a positive predictor for autonomous motivation in leisure time. Furthermore, as expected, autonomous motivation in leisure time was significantly positively related to the constructs of the theory of planned behavior, namely attitude, subjective norm, perceived behavioral control, and intention. Hence, the three constructs attitude, subjective norm, and perceived behavioral control represented partial mediators for the relationship between students’ autonomous motivation in leisure time and their intention to become physically active.

Furthermore, mindfulness was significantly positively related to autonomous motivation in physical education, autonomous motivation in leisure time, attitude, subjective norm, and perceived behavioral control (*p* < .05). Furthermore, autonomy support was directly related to mindfulness. As expected, mindfulness was neither directly related to intention nor to actual self-reported physical activity behavior. However, further serial mediation path analyses revealed that, as expected, mindfulness was indirectly related to the self-reported physical activity via attitude [subjective norm, and perceived behavioral control] and intention (*β_attitude_* = .01; *p* < .01; *β_norm_* = .01; *p* < .01; *β_control_* = .04; *p* < .01). Overall, Model 1b explained a considerable proportion of variance in all eight outcomes: autonomous motivation in physical education (*R*^2^ = .36), autonomous motivation in leisure time (*R*^2^ = .40), attitude (*R*^2^ = .26), subjective norm (*R*^2^ = .12), perceived behavioral control (*R*^2^ = .27), intention (*R*^2^ = .47), self-reported physical activity (*R*^2^ = .36), and mindfulness (*R*^2^ = .22).

**Figure 3:**
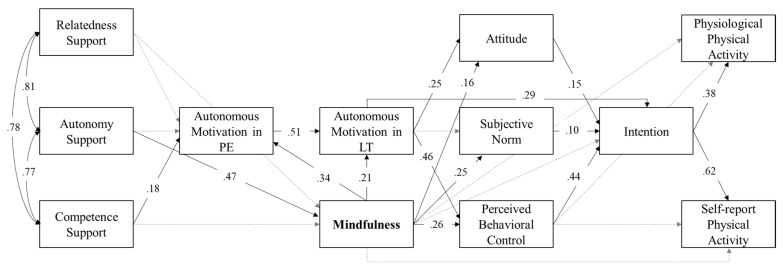
Path model (Model 2b) including mindfulness and physiologically measured physical activity. Model based on a sample size of *n* = 240 students. All calculated and standardized estimation paths are presented. Significant paths are depicted by solid arrows (*p* < .05), while non-significant paths are represented by dashed arrows (*p* > .05). PE = Physical education context; LT = Leisure time context.

In order to investigate the proposed assumptions beyond exclusive self-reporting measures, the inclusion of mindfulness into the trans-contextual model with physiologically measured physical activity is presented in Model 2b (Figure 3). Model 2a (without mindfulness) showed an acceptable model fit (please refer to Table 2), while Model 2b (additionally including mindfulness) showed a significantly improved and good model fit (*p* < .05; ΔCFI > .01). The results were comparable to those distinguishing Model 1a from Model 1b. Nevertheless, autonomy support, representing the first assumption of the trans-contextual model, was not significantly related to autonomous motivation in physical education in Model 2b. However, autonomy support significantly predicted mindfulness. For autonomous motivation in physical education, competence support was the only statistically significant predictor. Furthermore, as a second assumption of the trans-contextual model, autonomous motivation in physical education was positively related to autonomous motivation in leisure time. Autonomous motivation in leisure time, in turn, positively predicted attitude and perceived behavioral control, but not subjective norm. Except for the insignificant direct relation of perceived behavioral control and physical activity, all assumptions of the theory of planned behavior were confirmed. Attitude, subjective norm, and perceived behavioral control were directly related to intention, which, in turn, was directly related to self-report, as well as to the physiologically measured physical activity (MVPA per day).

Additionally, mindfulness was positively related to autonomous motivation in physical education and to autonomous motivation in leisure time, as well as to attitude, subjective norm, and perceived behavioral control. Consistent with Model 1b, mindfulness was, as expected, neither directly related to intention nor to physiological nor to self-reported physical activity. Nevertheless, mindfulness showed a significant indirect relation to physiological physical activity via attitude (subjective norm, and perceived behavioral control) and intention (*β_attitude_* = .01; *p* < .05; *β_norm_* = .01; *p* < .01; *β_control_* = .04; *p* < .01). Overall, Model 2b explained a considerable proportion of variance in all nine outcomes: autonomous motivation in physical education (*R*^2^ = .32), autonomous motivation in leisure time (*R*^2^ = .40), attitude (*R*^2^ = .12), subjective norm (*R*^2^ = .09), perceived behavioral control (*R*^2^ = .38), intention (*R*^2^ = .54), self-reported physical activity (*R*^2^ = .47), physiological physical activity (*R*^2^ = .11), and mindfulness (*R*^2^ = .14).

## 4. Discussion

As ideally ensuring the life-long learning of students represents a fundamental aim of education, the trans-contextual model [5] addresses this question, stating that autonomous motivation in school is transferred towards autonomous motivation in an out-of-school context, which is a key aspect in increasing overall physical activity rates in students both in and out of the schoolyard. In this sense, the aim of the present study was to verify if mindfulness, a concept which was found to be beneficial in education [38] can be conceptually integrated in the trans-contextual model, given the existing indications that mindfulness is related to the respective individual concepts of the model [44]. In this regard, the key question analyzed in the present study was whether the integration of mindfulness could improve the model fit of the trans-contextual model (i.e., whether mindfulness represents a beneficial concept to be implemented in schools in order to increase students’ overall physical activity rates).

### 4.1. Relationship between Mindfulness and SDT in the Trans-Contextual Model

Overall, our findings were in line with the assumptions of the trans-contextual model (please refer to Model 1a). However, in extension to the postulation that autonomy support directly influences autonomous motivation in physical education, we obtained mixed findings. In Model 1a, without mindfulness, we found competence support as well as autonomy support to exert a positive and significant direct influence, while the contribution of relatedness support was statistically insignificant. In line with the findings of Hagger and Chatzisarantis [5], autonomy support (e.g., enabling the students to choose an activity) proved to be important in the educational context in order to promote students’ autonomous motivation (Model 1a and 1b). However, in Model 2b (including mindfulness and physiological physical activity), the relationship between autonomy support and autonomous motivation remained statistically insignificant. On the other hand, and in congruence with Kiemer and colleagues [79], we found that competence support (e.g., providing individual goals) served as an important predictor for students’ autonomous motivation in school throughout all statistical models. The relationship between competence support and autonomous motivation was consistently statistically significant in all models with higher standardized path coefficients than autonomy support.

Furthermore, we were able to replicate the trans-contextual path between autonomous motivation in physical education and out-of-school autonomous motivation in leisure time. These results confirm that motivational regulation is transferable toward similar activities in different contexts [3]. Additionally, these findings highlight the importance of the motivational climate in the educational context, as teachers’ encouragement toward enhancing students’ autonomous motivation during physical education is likely to persist in leisure over time [6].

When specifically considering the association between mindfulness and basic need support, as well as the one between mindfulness and motivation, we found that solely students’ perceived autonomy support of the physical education teacher was positively related to their self-reported mindfulness. In this sense, we provided the first scientific indications that autonomy support is not only important to enhance the internalization of a behavior, thereby promoting autonomous behavior, but that the perceived autonomy support by the teacher is also positively related to mindfulness in students. This relationship was even found to transcend the path between autonomy support and autonomous motivation in physical education when referring to Model 2b, where physical activity was measured physiologically. Thus, mindfulness served as a full mediator between autonomy support and autonomous motivation. These results imply that teachers who enhance the autonomy of students’ self-regulated behavior, for instance by providing a choice of activities or encouraging them to ask questions, and ensuring they understand the goals of a given exercise, will foster students’ everyday life awareness that is, overall, non-judgmental (i.e., demonstrates mindfulness) [43].

In turn, higher mindfulness levels were found to be positively associated with the autonomous motivation of students not only in the context of physical education, but also for leisure time. The present results therefore confirm our assumption that a greater awareness of one’s values and goals will positively affect mindfulness to engage in autonomously motivated behaviors through the fulfillment of one’s need for autonomy. In this sense, mindfulness may promote engagement in self-regulated behaviors, that is, behavior that is congruent with one’s ‘true self’ and values (i.e., an autonomously motivated behavior) [42]. Thus, our results further underline the importance of promoting mindfulness in physical education, particularly in order to strengthen students’ awareness of their own values and goals to foster their internalization of behavior [80].

Interestingly, students’ perceived competence and relatedness support from the physical education teacher were not directly related to perceived mindfulness. As the support of both relatedness (i.e., fostering a feeling of respect and kindness) and competence significantly correlated with mindfulness (see Table 1), we infer that, in light of the trans-contextual model, our results further underline the key importance of autonomy for becoming aware of one’s own values and goals, in order to become autonomously motivated. Moreover, competence support seems to be a key factor in directly improving autonomous motivation, irrespective of mindfulness. In other words, competence support may foster autonomous regulation, but it may not necessarily increase students’ overall awareness of non-judgmentally being present in the moment.

### 4.2. Relationship between Mindfulness, SDT and TPB in the Trans-Contextual Model

Autonomous motivation in leisure time was directly related to attitude, subjective norm, and perceived behavioral control, which, in turn, positively predicted self-reported physical activity, mediated by intention. These findings support the assumptions of the trans-contextual model [1]. Put differently, autonomous motivation had a positive impact on the crucial components for the formation of a behavioral intention. Regarding the influence of autonomous motivation on subjective norm, we obtained mixed findings. In Model 1a and 1b, a small but significant positive effect was found. It needs to be stated that there is a disagreement in the scientific literature, as some scholars do not presume an association between the perceived subjective norm, representing a concept of social pressures, and autonomous motivation [81]. In contrast, several studies have presented evidence for a significant positive path between these two constructs [5]. The positive relationship between autonomous motivation and subjective norm found in some of our models might be interpreted as the respect or recognition of the wishes of the significant others as supportive or in accordance with self-endorsed values. In Model 1b, compared to Model 1a, however, the path coefficient decreased when adding mindfulness, and turned statistically insignificant in Model 2b.

### 4.3. Relationship between Mindfulness and TPB in the Trans-Contextual Model

In accordance with the theory of planned behavior, attitude, subjective norm, and perceived behavioral control were positively related to intention, which, in turn, positively affected actual physical activity behavior. With regard to the inclusion of mindfulness, our assumptions were met. Mindfulness was positively related to attitude, subjective norm, and perceived behavioral control. These results further underline the significant relationship between a higher situational awareness and the formation of an attitude toward a behavior. Notably, self-awareness has already been found to be relevant for achieving attitude–behavior consistency [50]. Similarly, mindfulness was positively related to perceived behavioral control. Apparently, if people are aware of their current situation, they may adapt to changes or challenges more quickly, leading to an experience of control [51]. Furthermore, our results support the assumption that mindfulness would be positively associated to subjective norm. In this sense, a greater overall awareness is likely to simultaneously increase the awareness of social norms and/or the pressures of significant others [54], which may eventually lead to prosocial responding [56]. Additionally, our assumption that mindfulness would neither directly affect intention nor actual behavior [57] could be confirmed. Our results indicate that the serial mediation paths of mindfulness on attitude, perceived behavioral control, and subjective norm on intention, and, finally, on actual physical activity behavior, were significant.

A particular strength of the present study is the fact that our assumptions were not only met for the self-reported measure of physical activity, but also for physiologically measured physical activity. As expected, self-reported and physiologically measured physical activity generally did not correlate to a great extent (*r* = .31 in our study; comparable to the results of Hagstromer and colleagues [58]), especially when considering that both variables were measuring the same construct. Nevertheless, our proposed model including mindfulness into the trans-contextual model could be confirmed for both constructs, which further strengthens the trans-contextual model. As our mindfulness-including Models 1b and 2b revealed an improved overall model fit, compared to their counterparts without mindfulness, this further highlights the importance of our suggestion to take into account the beneficial role of mindfulness in the educational context.

### 4.4. Implications for Education

Our results suggest that autonomy support is not only an important factor to foster autonomous motivation, but also to support the development and maintenance of mindfulness. As the process of internalizing a healthy behavioral regulation requires cognitive mechanisms such as, for example, awareness [43], the present results underline that mindfulness is directly related to autonomous motivation. In this sense, a non-judgmental awareness of the present moment may increase a more objective view of one’s own behavior and values, which, through self-reflection, helps to bring values and needs into congruence with one’s actual behavior. Thus, mindfulness is crucial in order to be aware if one is consistently acting with one’s authentic self [43]. In sum, based on our overarching conceptual findings that mindfulness is related to motivation both in school and beyond the educational setting, this specifically stresses our recommendation to implement such interventions aimed at fostering mindfulness in schools on a large scale.

With regard to practical examples for implementing mindfulness in education, as described, for instance, in Saltzman and Goldin [82], adequate mindfulness exercises (e.g., breathing exercises, the 5-4-3-2-1 exercise, a short a priori mindfulness reflection on “what is my personal goal for today?”, or body scan, etc.), ought to be made available to students, as they result in increased mindfulness rates, while simultaneously being experienced as meaningful for their everyday life. Ultimately, mindfulness-based interventions in schools represent promising tools, particularly for improving cognitive performance as well as students’ resilience in the face of stress [38].

### 4.5. Limitations and Suggestions for Future Research

One limitation of the present study is that, at the time of the data collection period, no questionnaire to assess the students’ three distinct constructs of basic need support had been empirically validated and published. However, we found good psychometric properties using and adapting the scales by Standage and colleagues [62]. In relation to this, in the present extensive study, the number of items for each scale was considerably low. Nevertheless, all scales showed a good reliability and a larger questionnaire would have been unreasonably time consuming for the children, and difficult to complete in one session.

With regard to the mindfulness scale, the FMI [67] was used. However, it needs to be noted that, usually, the Mindfulness and Attention Awareness Scale (MAAS) [42] has been used in order to analyze the relationship between autonomous motivation and mindfulness. The MAAS differs from the FMI insofar as it focuses solely on (self-) attention and awareness, with exclusively negatively formulated “mindlessness” items (e.g., “I rush through activities without being really attentive to them”) [83]. The FMI is, similar to the MAAS, a unidimensional scale. However, it includes a more Buddhistic approach to measuring mindfulness, thereby exceeding the MAAS in the aspects of “non-judgement, accepting attitude, dis-identification, insightful understanding, or an attitude of having no specific goals” [67] (p. 1545). Both instruments were found to be reliable and valid [42,83]; however, in order to analyze mindfulness (and not mindlessness) in light of the non-judgmental awareness of the present moment, the FMI was used in the present study, which proved to be related to autonomous motivation in the physical education and leisure time context.

In addition, the complete autonomous motivational regulation scale of SDT [7] could not be addressed. The integrated regulation subscale that represents the most internal motivational regulation of the extrinsic motivation continuum is considered to be very similar to intrinsic motivation, as the internal values of the behavior in question are defined as being completely integrated into the sense of self and wholly volitionally performed [84]. As an integrated motivation form may exert a differentiated role within the trans-contextual model, it should be inclusively measured in future studies. In particular, as integrated regulation requires self-reflection and awareness to integrate personal values [8], mindfulness may play a specific role regarding this motivational regulation type. However, to the best knowledge of the authors, the scale in question had not yet been empirically validated for the physical education context at the time of data collection.

A further limitation of the present study and suggestion for future research would be to investigate an extension of the trans-contextual model beyond the context of physical education, in order to verify the impact of mindfulness at a more general educational level. Finally, as we provide a first indication that mindfulness can be integrated into the trans-contextual model, we suggest that upcoming research should focus on longitudinal or intervention studies in education, in order to confirm the supposed directions of the path models.

## 5. Conclusions

In conclusion, we obtained profound scientific results indicating that mindfulness is applicable to the trans-contextual model and that it provides an important tool for targeted interventions to be implemented in class. More specifically, mindfulness was significantly predicted by the teacher’s support of autonomy and directly related not only to autonomous motivation in school, but also to the students’ autonomous motivation in leisure time. Furthermore, mindfulness appeared to be directly related to the basic cognitive concepts of the theory of planned behavior, indicating that mindfulness is indirectly related to actual behavior, mediated by the attitude toward the specific behavior, as well as by perceived behavioral control. The present study thereby replicates and extends the findings of the trans-contextual model not only based on self-report measures, but also using physiologically measured physical activity. As mindfulness has proven to be an important extension of this model for both self-reported and physiologically assessed physical activity behavior, we strongly recommend that interventions aimed at fostering students’ mindfulness receive greater attention in educational settings. Ultimately, in coming back to the initial question that addressed the need for education to also exert a significant influence on students’ everyday life health behaviors, our results suggest that mindfulness is an important aspect that substantially extends the trans-contextual model. Thus, mindfulness serves as a promising exemplary competence for the desired further encouragement of knowledge and beneficial skills learned in school to become integral parts of the everyday lives of students.

## Figures and Tables

**Table 1:** Descriptive statistics and correlation matrix of the study variables.

	*M*	*SD*	1.	2.	3.	4.	5.	6.	7.	8.	9.	10.	11.	12.
1. Relatedness Support	5.34	1.13	–											
2. Autonomy Support	4.97	1.10	.82 *	–										
3. Competence Support	5.35	1.21	.82 *	.82 *	–									
4. Autonomous Motivation (PE)	5.12	1.31	.50 *	.53 *	.54 *	–								
5. Autonomous Motivation (LT)	5.18	1.29	.38 *	.39 *	.41 *	.59 *	–							
6. Attitude	5.63	1.23	.23 *	.24 *	.25 *	.39 *	.50 *	–						
7. Subjective Norm	4.04	1.41	.20 *	.25 *	.21 *	.25 *	.24 *	.18 *	–					
8. Perceived Behavioral Control	5.23	1.37	.30 *	.31 *	.31 *	.33 *	.47 *	.39 *	.23 *	–				
9. Intention	4.25	1.66	.26 *	.27 *	.28 *	.39 *	.61 *	.48 *	.23 *	.54 *	–			
10. Self-report Physical Activity	3.20	1.97	.17 *	.17 *	.19 *	.23 *	.44 *	.34 *	.10 *	.39 *	.60 *	–		
11. Physiological Physical Activity ^a^	47.47	19.60	−.01	−.01	−.04	.12	.22 *	.16 *	.14 *	.16 *	.33 *	.31 *	–	
12. Mindfulness	4.66	.90	.40 *	.46 *	.41 *	.44 *	.47 *	.29 *	.34 *	.41 *	.34 *	.21 *	.14 *	–

*Note*. * *p* < .05; PE = Physical education context; LT = Leisure time context. ^a^ sample size for the correlations of physiological physical activity (MVPA in minutes per day) and other variables was *n* = 240, all other correlations were performed with a sample size of *N* = 1877.

**Table 2:** Model fit indices of the four path models.

	*N*	*df*	*χ* ^2^	RMSEA	RMSEA 90%	SRMR	CFI	TLI
Model 1a without mindfulness	1877	29	338.519 *	.075	[.068; .083]	.073	.968	.950
Model 1b with mindfulness	1877	29	210.951 *	.058	[.051; .065]	.038	.983	.967
Model 2a without mindfulness	240	36	86.279 *	.076	[.056; .097]	.087	.959	.937
Model 2b with mindfulness	240	36	66.478 *	.060	[.036; .082]	.061	.977	.958

*Note.* * *p* < .05.

## Data Availability

Not applicable.
